# Supplementary material for: A Pilot Study on the Inter-Operator Reproducibility of a Wireless Sensors-Based System for Quantifying Gait Asymmetries in Horses
Source: Sensors (Basel). 2022 Dec 6;22(23):9533. doi: 10.3390/s22239533 (PMC9740227; doi:10.3390/s22239533)
Supplement: Supplementary file 1 [file sensors-22-09533-s001.zip › sensors-1972996-supplementary.pdf]

Supplementary materials:

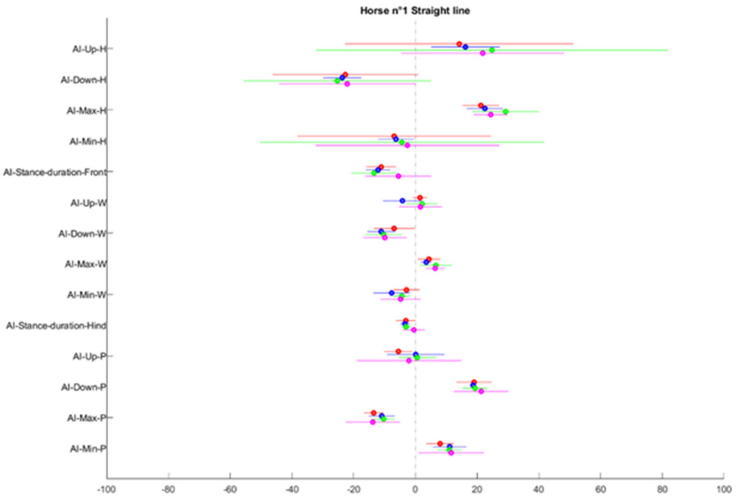

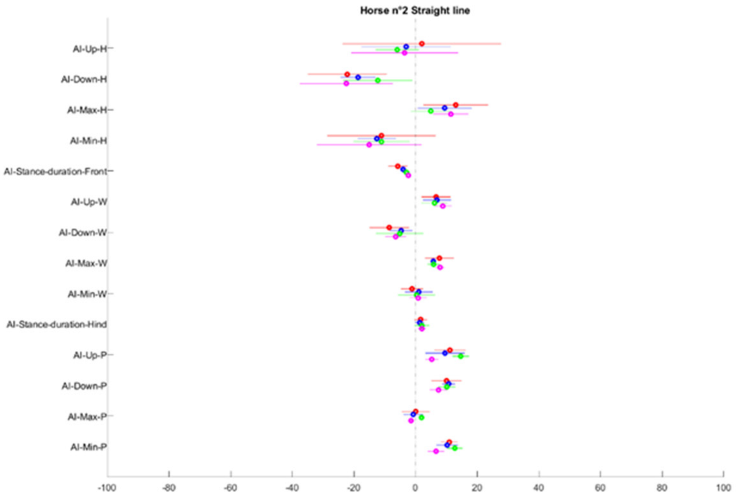

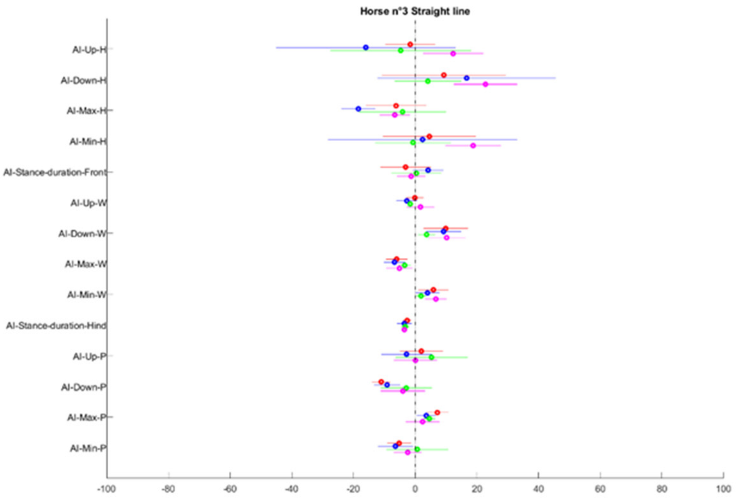

Figure S1. Asymmetry indexes (mean and SD) measured on the head (-H), the withers (-W) and the

pelvis (-P) of three horses trotting **on a straight line** by four operators (operator A: red dot, operator B: blue dot, operator C: green dot and operator D: magenta dot) repeating 12 times the measurements for each horse, after systematically repositioning the sensors at each recording.

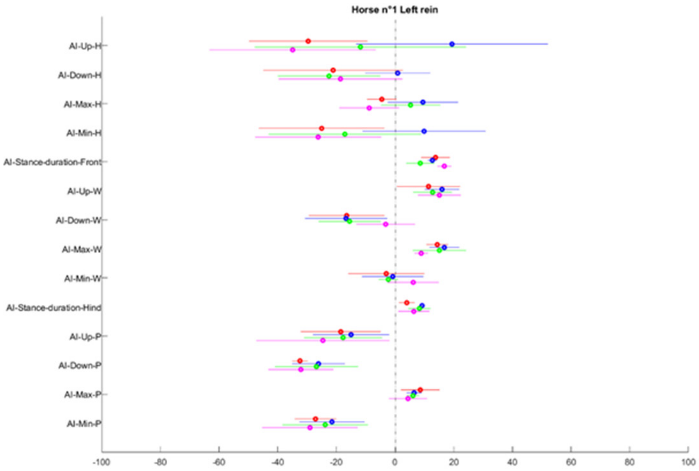

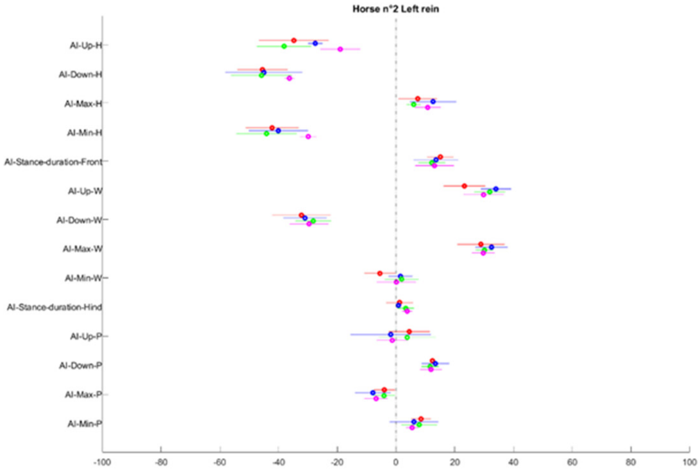

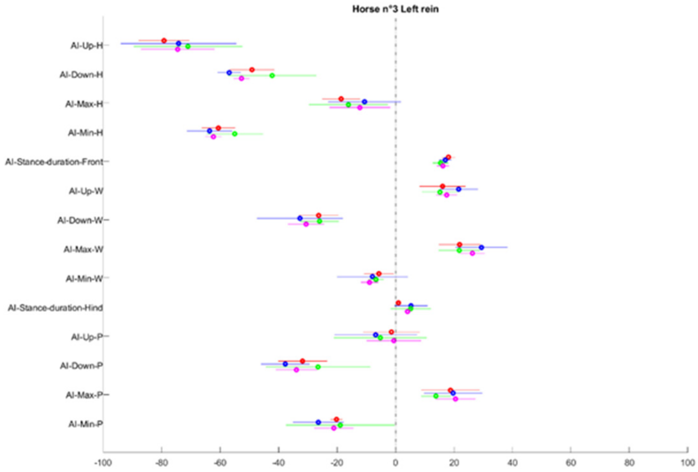

Figure S2. Asymmetry indexes (mean and SD) measured on the head (-H), the withers (-W) and the pelvis (-P) of three horses trotting **on a left rein circl**e by four operators (operator A: red dot,

operator B: blue dot, operator C: green dot and operator D: magenta dot) repeating 12 times the measurements for each horse, after systematically repositioning the sensors at each recording.

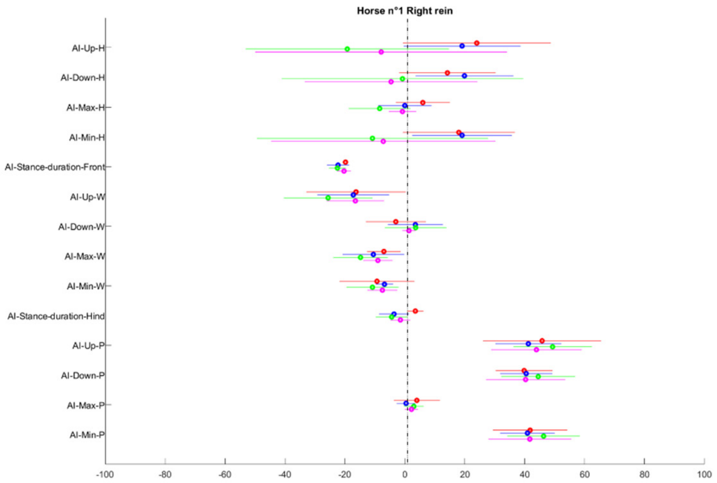

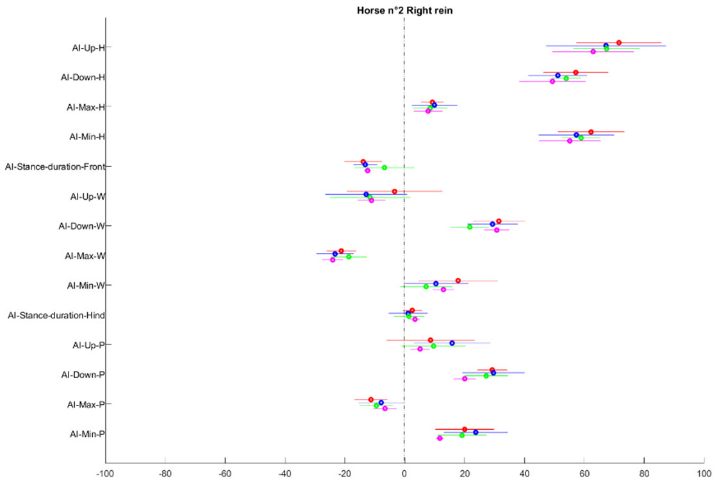

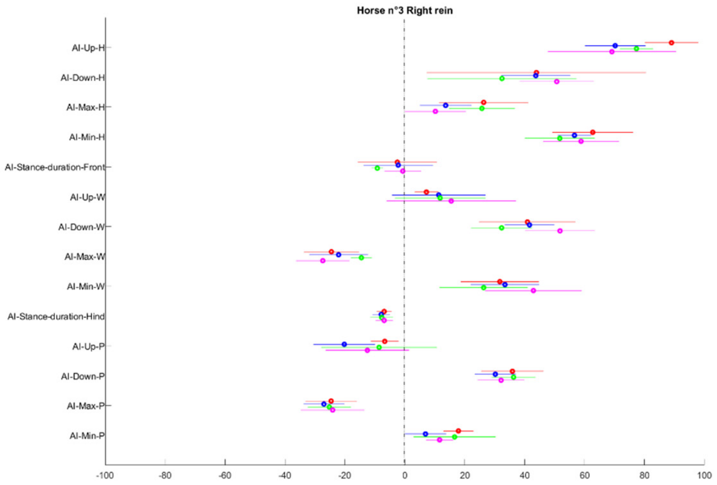

Figure S3. Asymmetry indexes (mean and SD) measured on the head (-H), the withers (-W) and the pelvis (-P) of three horses trotting **on a right rein circl**e by four operators (operator A: red dot,

operator B: blue dot, operator C: green dot and operator D: magenta dot) repeating 12 times the measurements for each horse, after systematically repositioning the sensors at each recording.
